# Changes in Fluorescence of Aquatic Dissolved Organic Matter Induced by Plastic Debris

**DOI:** 10.3390/ma18071602

**Published:** 2025-04-01

**Authors:** Cristina L. Popa, Simona I. Dontu, Dan Savastru, Elfrida M. Carstea

**Affiliations:** National Institute of R&D for Optoelectronics, INOE2000, Atomistilor 409, 077125 Magurele, Romania; cristina.popa@inoe.ro (C.L.P.); dsavas@inoe.ro (D.S.)

**Keywords:** fluorescence spectroscopy, plastic, dissolved organic matter, water quality

## Abstract

Water contamination with plastic materials represents one of the most pressing environmental problems that the modern world is facing. In this context, the present paper aims to investigate the influence of fluorescent dissolved organic matter (FDOM) released by plastic materials on the aquatic bacterial fraction and evaluate the efficiency of fluorescence spectroscopy in identifying plastic FDOM in freshwater. To this purpose, river and tap water samples were contaminated in a controlled manner in the laboratory, and the water quality parameters and bacterial occurrence for these samples were determined using standard physico-chemical characterization methods: fluorescence spectroscopy, dynamic light scattering, and flow cytometry. The results revealed that plastic debris influenced the dissolved-particulate organic matter continuum, also affecting bacterial cell proliferation in both the river and tap samples. The study highlights that the impact of plastic FDOM on bacterial proliferation should not be taken lightly, while fluorescence spectroscopy proved to be an effective method for identifying the presence of plastic FDOM in water samples of various origins.

## 1. Introduction

Large amounts of plastic waste are continuously released into the environment, becoming a growing global threat [1]. Estimates show that in 2016, 11% of the global plastic waste reached aquatic ecosystems [2]. Large plastic waste enters freshwater systems through natural processes (such as wind or surface runoff) or through direct dumping [3]. Plastic waste is likely to accumulate in the system because most of the plastic polymers are non-biodegradable, and the main degradation mechanisms in water (solar radiation and thermal oxidation) are relatively slow in degrading plastic [1]. These mechanisms can break down plastic into small fragments and microplastics (particles < 5 mm), which may be ingested by aquatic animals, leading to starvation or changes in behaviour, reproduction, and growth [3]. Moreover, several additives can leach into the water with potential toxic effects on aquatic life [1,3,4]. Recent studies show that plastic can leach fluorescent dissolved organic matter (FDOM) [5,6,7,8,9,10,11,12,13]. FDOM is a heterogeneous mixture of compounds originating from natural and anthropogenic sources, and is ubiquitous to aquatic systems [14,15]. Few studies show that plastic-leached FDOM affects microbial communities [10,16,17,18]. However, studies that focus on commercially available plastic products have been scarce [7,16,19,20]. Commercial plastic products may turn into plastic litter and are likely to remain intact for decades [3] until they decompose into smaller and smaller fragments. Moreover, most of these studies focused only on the marine environment or engineered water systems. In order to expand knowledge on plastic FDOM, the aim of this study was to evaluate the release of FDOM from large plastic fragments—polystyrene and low-density polyethylene—into tap and river water samples with effluent intake. In particular, the study aimed to (1) assess the changes in FDOM leached from plastic in water samples, (2) determine the influence of plastic FDOM on the aquatic bacterial fraction, and (3) analyse the potential of using specific fluorescence peaks as indicators of plastic FDOM in freshwater.

## 2. Materials and Methods

### 2.1. Sample Preparation

Approximately 10 L of river samples were collected from the Ciorogarla River (Magurele, Romania, 44.33° N, 26.05° E) after a wastewater treatment plant release point. Ciorogarla Basin has a size of 149 km^2^ and collects wastewater effluents (with 12,500 PE) and untreated water that is illegally released into the river. It crosses mostly an agricultural area and flows into the Arges River. Ciorogarla samples were collected in February 2021, after a week free of precipitation. Also, 10 L of tap water were sampled after allowing the water to run for a few minutes. The samples were homogenized and divided into 7 identical samples in 1 L glass bottles. Sampling bottles were pre-cleaned with 2% RBS solution and thoroughly rinsed with distilled water. Bottles were also rinsed with the sampled river and tap water prior to collection. The samples were transported to the laboratory within 1 h of collection and allowed to reach room temperature before the addition of plastic debris. Samples were not filtered to preserve all DOM fractions.

Commercially available LDPE sandwich bags and PS coffee cup lids were used in the study. Ten PS coffee cup lids (11.90 g) and ten LDPE food bags (7.77 g) were added to each sample. Three replicates were prepared for each sample (3× River + PS, 3× Tap + PS, 3× River + LDPE, and 3× Tap + LDPE). One control sample (with no added plastic) was prepared for the tap and river sample sets. The samples were kept for 35 days at room temperature under normal light conditions (not under direct sunlight). The samples were gently shaken before each set of measurements. 10–15 mL of sample was extracted from each bottle for measurements in order to reduce the impact on the overall volume of the sample.

### 2.2. Standard Water Quality Parameters

Total organic carbon (TOC) was measured using the PF-12Plus photometer (Macherey-Nagel, Düren, Germany) and water quality testing kits from the same manufacturer. The measurements were performed according to the kit’s manufacturer instructions.

### 2.3. Dynamic Light Scattering

Dynamic light scattering (DLS) measurements were made using Zetasizer Nano ZS90 equipment (Malvern Instruments, Malvern, UK). The ZS90 is equipped with a 50 mW laser, having a wavelength of 532 nm, and the measurements were made at an angle of 90°. The narrow band filters that are part of the instrument improve the signal for fluorescent samples. Hydrodynamic size distribution measurements were performed at a temperature of 22 °C, with an equilibration time of 120 s. For each measurement, a disposable polypropylene cuvette was used. Each sample was measured in triplicate, without delays between measurements.

### 2.4. Flow Citometry

The flow cytometry (FCM) measurements were performed with a BD Accuri C6 Plus cytometer (BD Biosciences, San Jose, CA, USA), equipped with two lasers (blue—488 nm and red—640 nm), two scatter detectors, and four fluorescence detectors. In order to isolate the bacterial population, a staining protocol was applied for all of the samples. SYBR Green I (Sigma-Aldrich, St. Louis, MO, USA) and Propidium (PI) dyes (Sigma-Aldrich, St. Louis, MO, USA) were used according to the Eawag method [21] for assessing water quality. 1 mL of each sample was stained with 10 µL of SYBR Green I (10,000× dilution of DMSO stock solution) and 1 µL of PI (final concentration of 0.3 mM). After staining, each sample was kept in the dark at around 35 °C for 10 min. For each measurement, the following parameters were used: 50 µL of a volume of 500 µL, flow rate of 11 µL/min, core size of 5 µm, and threshold of 800 on FL1-H. For each sample, two-dimensional FL1-A (emission filter 533/30 nm) vs. FL3-A (emission filter > 670 nm) log-scale density plots were recorded. In order to separate low nucleic acid content (LNA) from high nucleic acid content (HNA), a BD Accuri C6 software (Version 1.0.23.1) template provided by BD Biosciences was used. Thus, the data acquisition was gated on the PFL1-A vs. FL3-A plots using a lower limit of 2000 on FL1. The bacterial cell gate included LNA and HNA bacteria. LNA bacteria are small and stain weakly with SYBR Green I, while HNA bacteria are large and stain brightly with SYBR Green I [21].

### 2.5. Fluorescence Spectroscopy

Fluorescence excitation–emission matrices (EEMs) (Figure S1) were recorded with a FP8200 spectrofluorometer (Jasco Corporation, Tokyo, Japan) using the following parameters: λ_exc/em_ = 200–500/240–550 nm, step 1 nm, slit 5 nm, integration time 20 msec. Raman spectra were recorded to check the stability of the instrument. Raman values varied between 36.92 a.u. and 38.53 a.u. (median value 37.34 a.u.). No inner filter correction was applied to the EEMs. The TOC value of the river sample was <20 mg/L and that of the tap water sample was <2 mg/L. According to past studies [22], the inner filter effect is unlikely to affect the fluorescence spectra of samples with TOC values below 25 mg/L. Fluorescence peaks were extracted from the blank corrected and Raman corrected EEMs [23] using the peak-picking method [24]. The following peaks were extracted: peak B (λ_exc/em_ = 230–240/300–310 nm), peak T_230_ (λ_exc/em_ = 230–240/326–350 nm), peak T_280_ (λ_exc/em_ = 270–282/328–350 nm), peak M (λ_exc/em_ = 290–310/370–420 nm), peak A (λ_exc/em_ = 230–238/400–424 nm), peak C (λ_exc/em_ = 310–350/400–450 nm). In addition, peaks described by Lee et al. [7] for plastic-derived FDOM were extracted as follows: H (λ_exc/em_ = 290/405 nm), L (λ_exc/em_ = 230/405 nm), P (λ_exc/em_ = 270/305 nm). Finally, peaks for optical brighteners were extracted according to Gandhimathi et al. [25]: 1,4-diphenylbutadiene (DPBD) (λ_exc/em_ = 330/375 nm), Uvitex-OB (UVX) (λ_exc/em_ = 375/425 nm), benzophenone (BPN) (λ_exc/em_ = 252/418 nm). The biological index (BIX), humification index (HIX), and the F450/500 index were calculated as recommended by Huguet et al. [26], Zsolnay et al. [27], and McKnight et al. [28], respectively.

### 2.6. Statistical Analysis

Statistical significance was determined using the Kruskal-Wallis H test, with a Nemenyi follow-up test (*p* < 0.01) [29]. The distribution of the data was considered non-normal (Shapiro-Wilk test, W = 0.995–0.656, *p* < 0.05).

## 3. Results and Discussions

### 3.1. Dynamic Light Scattering Data

DLS measurements revealed that for the control river sample, the average particle size doubled after the first 7 days of storage, increasing 4-fold by day 28 (Figure S2). The control tap water sample displayed an increase of only 31%, followed by a slow increase towards day 21 and a sudden decrease towards the end of the storage period. DOM polymers were shown to assemble into microgels up to an equilibrium size of 6 µm, which is the maximum size of the average DOM polymer chain length [30]. The tap water sample with added plastic displayed a different trend to the control sample, decreasing slightly towards day 35; however, a similar behaviour was observed between the LDPE and PS treatments. In the case of the river samples, only the PS sample showed increasing average particle size up to day 14, and decreased towards the end of the experiment. However, the River + LDPE sample displayed an almost constant average particle size during the entire period. Potentially due to the high surface size of LDPE bags compared to the fragments of the PS coffee cup lids, more particles adhered to LDPE surface. Thus, large plastic pieces may lead to a reduction in microgels as particles adhere to the plastic surface. These results potentially indicate that large plastic debris can disrupt the DOM to particulate organic matter (POM) continuum in a freshwater environment.

### 3.2. Impact of Plastic on Bacterial Cells Abundance and Behaviour

**Figure 1:**
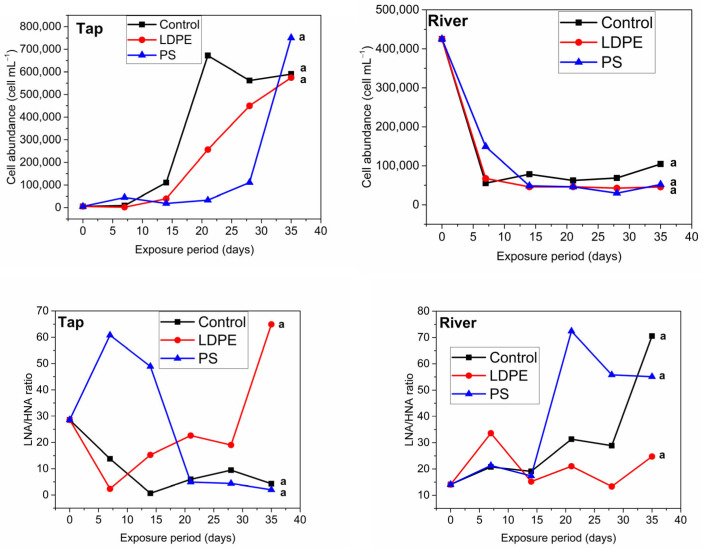
Bacterial cell count and the LNA/HNA ratio for the river (control, River + LDPE, River + PS) and tap samples (control, Tap + LDPE, Tap + PS). The groups indicated with the same letter show that they are not significantly different (*p* < 0.01). HNA—high nucleic acid, LNA—low nucleic acid.

Cell abundance in the control tap sample increased slowly towards day 14 and showed a six-fold increase towards day 21 (Figure 1). For the Tap + LDPE sample, the cell abundance followed the same trend as the tap control sample but had lower values compared to control, while for the Tap + PS sample, cell abundance remained almost constant until day 28 and increased seven-fold towards day 35. The difference between datasets was not significant. However, these minor changes may indicate that plastic slightly delays or decreases cell proliferation. For example, Chen et al. [16] found that FDOM leached from plastic was toxic to a freshwater luminescent bacterium (Vibrio qinghaiensis Q67), while Mohamed et al. [31] showed that a plasticizer (di(2-ethylhexyl) terephthalate), found in polyvinyl chloride films can have a toxicity effect on gram positive bacteria (Rhodococcus ruber). Thus, substances leached from plastic can be toxic to certain bacteria. All of the river samples displayed the same decreasing trend throughout the experiment (Figure 1) and no significant impact from plastic on cell abundance was found.

The ratio between LNA and HNA may show the transition from a dormant to an actively growing bacterial community due to changes in the nutrient level in water systems [32,33]. While both LNA and HNA cells may be metabolically active, LNA cells may become dormant to withstand limited nutrient concentrations. HNA cells are more dynamic and sensitive to environmental changes compared to LNA cells [32,33]. Sharuddin et al. [32] suggested that an increase in HNA cells may come from LNA cells that are dormant in a limited nutrient environment, but which, in favourable conditions, may become fast growing cells with high DNA content. Consequently, these will be detected as HNA cells. The decrease in the LNA/HNA ratio for the control tap sample indicated an increase in HNA cells, potentially due to an increase in bacterial growth from storage at room temperature [34]. After 14 days of storage, the LNA/HNA ratio in the control sample showed a slight increase, potentially due to nutrient depletion. For the Tap + PS sample, the LNA/HNA ratio doubled within the first week of exposure, followed by an abrupt decrease towards day 21 to the level observed at the control sample. Potentially, in the first weeks of exposure to PS, bacterial growth was inhibited, while later the organic carbon that leached from plastic acted as a food source for microorganisms, leading to their activation. An abrupt decrease in the LNA/HNA ratio was observed in the case of the Tap + LDPE sample, suggesting that substances leaching from LDPE favoured bacterial growth immediately after exposure. Our results are supported by Romera-Castillo et al. [10], who showed that dissolved organic carbon (DOC) leaching from LDPE is consumed by microbes in water almost immediately after incubation. However, our results contradict the study of Harshvardan & Jha [35], who found that the metabolic activity of certain bacteria increased after 14 days of incubation with polyethylene. Although more studies on tap water are needed, the current results may have greater implications with regard to drinking water sources. As substances leaching from plastic act as food source and may increase bacterial growth, more intense water treatment may be needed for drinking water supply, increasing the cost of treatment processes. Left untreated, contaminated water may have a larger impact on the ecosystem and human health.

The river samples showed different trends compared to the tap samples with regard to the LNA/HNA ratio (Figure 1). The control sample showed that bacteria were relatively metabolically active until day 28, when a spike in the LNA/HNA ratio indicated a sudden drop in HNA cell content relative to LNA cells. The proportion of HNA cells dropped earlier in the River + PS sample compared to control sample. Previous studies showed that substances leaching from PS can be toxic and inhibit the growth of water microorganisms [36,37]. The River + LDPE sample showed a relatively constant LNA/HNA ratio throughout the experiment. These findings potentially indicate that microorganisms preferentially consume the existing food sources in nutrient rich waters and using leached DOC when nutrients become scarce in the environment. Also, active cells in the river sample may have adhered to plastic surfaces forming biofilm, which may have led to a constant LNA/HNA ratio in the LDPE samples. Urbanek et al. [38] and Oberbeckmann et al. [39] have investigated the formation of biofilm on PS and polyethylene, demonstrating that in cold marine water conditions, after a period of incubation of 2 weeks, the microplastics were covered in microbial assemblages. Our results show that plastics’ potential to disrupt microbial activity in water systems must not be underestimated considering the quantity of plastics that reach aquatic systems, and it warrants further analysis with a larger sample size compared to this study.

### 3.3. Plastic-Derived Fluorescent Dissolved Organic Matter

**Figure 2:**
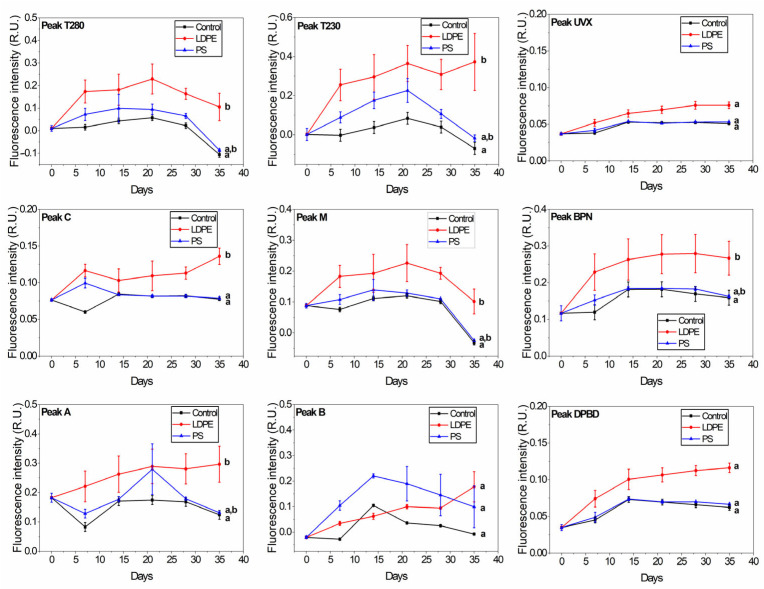
Changes in the release of plastic-derived FDOM in tap water samples (control, Tap + LDPE, Tap + PS). The peaks correspond to the protein-like fraction (peaks T230, T280 and B), humic-like matter (peaks A, C and M), and optical brighteners-like matter (peaks UVX, BPN and DPBD). The groups indicated with the same letter show that they are not significantly different (*p* < 0.01).

The EEMs showed the presence of nine peaks corresponding to the protein-like fraction (peaks B, T230 and T280), humic-like matter (peaks A, C and M), and optical brighteners-like matter (peaks BPN, DPBD and UVX). Tap water samples displayed significantly higher fluorescence intensity (*p* < 0.01) at all of the peaks when LDPE was added compared to control (Figure 2). Jacques and Poller [40] showed that the fluorescence of LDPE is due to α,β-unsaturated carbonyl compounds resulting from the oxidation of polymers. At the Tap + LDPE sample, peaks A and C showed a relatively continuous increase towards the end of the exposure period. Yan et al. [41] found that humic-like substances are preferentially released from MP-DOM with UV aging. Thus, the daily light conditions that the samples were exposed to in our study may have favoured the steady release of peaks A and C.

PS did not significantly increase the quantity of FDOM compared to the control sample, except for peak B. Peak B from the Tap + PS samples increased continuously for 14 days, followed by a drop towards day 21, arriving almost to the initial value by day 35. Peak B from the Tap +LDPE sample showed only a slight increase compared to the control, followed by a sudden increase from day 28 to day 35. Previous studies also proved that plastic releases FDOM in water in the peak B region [5,6,8,10,12,20,42,43,44] irrespective of plastic type. The fluorescence of this peak may originate from residual monomers and oligomers, and plastic additives that can leach from PS and LDPE. These substances were found to migrate from plastic packages [1]. Styrene oligomers were shown to leach from PS food packaging and coffee cups after contact with distilled water at room temperature [45,46,47]. Styrene displays fluorescence in the peak B range (λ_exc/em_ = 255/305 nm) [48]. Phenol was also found to migrate from PS in a solution of 20% ethanol [49]. Phenol displays fluorescence at ~290 nm when excited with 270 nm [50]. Zhao et al. [51] found that phenol has a peak at 304 nm; however, in a different solution. Peak B fluorescence may also indicate a contribution from the bisphenol A (BPA) additive. BPA migrates from PS and LDPE food containers into water [52,53] and displays fluorescence at around λ_excn/em_ = 225/311 nm [8].

Tap + LDPE samples displayed a significantly different fluorescence signal compared to the control for the BPN peak (*p* < 0.01), and a slight, but non-significant difference for the DBPD and UVX peaks. DPBD, UVX, and BPN are optical brighteners that mask the yellow appearance of weathered plastic and provide a robust colour [25,54]. DPBD, in particular, was found to leach from LDPE plastic packages [25]. Optical brighteners absorb light in the range of 320–400 nm and emit between 400–480 nm [54]. The DPBD-like peak increased 3-fold from day 0 to day 35 in the case of the Tap + LDPE sample, and it was almost double in intensity compared to the Tap + PS and control samples. UVX-like and BPN-like peaks registered for the Tap + LDPE sample increased approximately 2-fold from day 0 to day 35. The lack of fluorescent DOM released from PS for these peaks may have been caused by the presence of TiO_2_. This component is added to plastic for a bright white appearance [54]. TiO_2_ exhibits an absorbance peak at 354 nm [55] and fluorescence emission at ~700 nm [56]. TiO_2_ competes with optical brighteners for UV light needed in order to function [54]. In addition, it may absorb the fluorescence emitted by peak C components.

**Figure 3:**
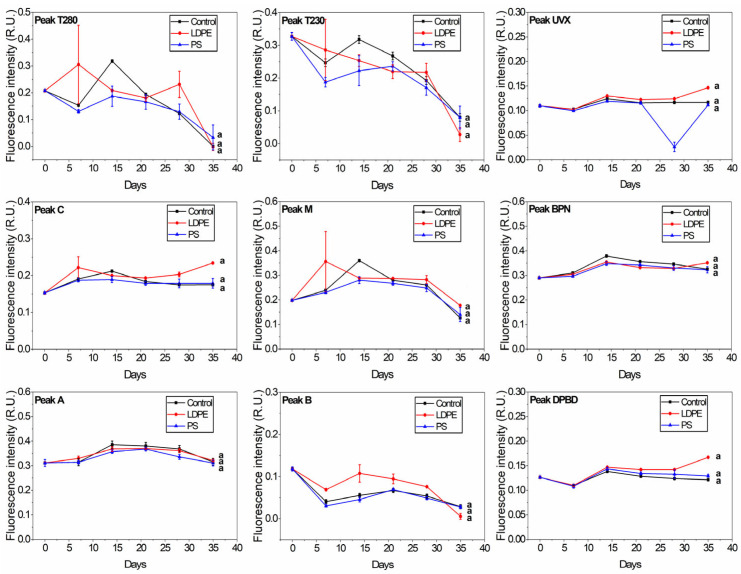
Changes in the release of plastic-derived FDOM in river water samples (control, River + LDPE, River + PS). The peaks correspond to the protein-like fraction (peaks T230, T280, and B), humic-like matter (peaks A, C, and M) and optical brighteners-like matter (peaks UVX, BPN, and DPBD). The groups indicated with the same letter show that they are not significantly different (*p* < 0.01).

River samples showed no significant difference between control, PS, and LDPE at any of the peaks (Figure 3). In general, the fluorescence of the protein-like peaks (B, T230, and T280) decreases with storage over long periods [57] due to bacterial degradation and humic-like matter formation. Peaks B and T230 degradation was slightly lower in the River + LDPE sample towards day 35. Oberbeckmann et al. [39] found differences between PE and PS microbial assemblages depending on the aquatic environment. They observed that differences were low with an increase in nutrient levels. This may explain the differences in FDOM between the river and tap samples in response to plastic addition. Moreover, DOC leached from plastics may have been used as a food source by the microbial fraction in the river samples, as shown by the flow cytometry data.

Most of the fluorescence indices did not show significant differences between the control and plastic treatments for the tap and river samples (Figure S3). A significant difference (*p* < 0.01) was observed at the T/C ratio between the Tap + LDPE sample and the other two samples. HIX values were significantly different (*p* < 0.01) for Tap + PS compared to the other samples. This highlights the influence of phenol-like components at peak T fluorescence, in particular those released by LDPE. Fluorescence index F450/500 showed significant differences between River + LDPE and the other samples (Figure S3). This difference was not observed by analysing the individual peaks—DPDB, UVX, and BNP (Figure 3)—which suggests that other compounds may be released by LDPE.

## 4. Conclusions

This study indicated that plastic debris changed the characteristics and composition of aquatic FDOM. The increase in fluorescence intensity suggested the migration of residual monomers, oligomers, and optical brighteners from plastic debris into water samples. Leaching plastic substances did not significantly affect cell abundance, but changed the content of LNA and HNA cells. In samples with low organic matter content, such as tap water, the substances from LDPE may have acted as food sources, activating dormant cells, while PS may have inhibited bacterial growth in the first weeks of exposure. In river water samples, the data suggested that microorganisms may have preferentially consumed existing food sources. They potentially switched to LDPE-leached substances or became inactive in the case of PS samples when the other food sources were depleted. The data also indicated that FDOM may have adhered to the surface of plastic, in particular to LDPE. The changes in FDOM were significantly different in the samples with low background FDOM, such as the tap water samples. In these samples, LDPE released significantly higher quantities of FDOM compared to PS. In the case of the river samples, no significant differences were observed between the control, PS, and LDPE samples; thus, a full analysis of fluorescence peaks and indices was needed to identify the changes in FDOM induced by plastic debris in water.

## Data Availability

The original contributions presented in this study are included in the Supplementary Materials. Further inquiries can be directed to the corresponding authors.

## Supplementary Materials

The following supporting information can be downloaded at: https://www.mdpi.com/article/10.3390/ma18071602/s1, excel file “All data” containing all used data and “Supplementary material” containing Figure S1: Fluorescence excitation-emission matrices for control day 0, control day 14, Tap + LDPE day 14, River + LDPE day 14, Tap + PS day 14, River + PS day 14; Figure S2: The average hydrodynamic size distribution for the river and tap water samples. Parameters that present the same letter are not significantly different at *p* < 0.01; Figure S3: Changes in fluorescence index values for tap and river samples exposed to PS and LDPE. The groups indicated with the same letter indicate that they are not significantly different (*p* < 0.01).
