# Supplementary material for: Changes in Fluorescence of Aquatic Dissolved Organic Matter Induced by Plastic Debris
Source: Materials (Basel). 2025 Apr 1;18(7):1602. doi: 10.3390/ma18071602 (PMC11990298; doi:10.3390/ma18071602)
Supplement: Supplementary file 1 [file materials-18-01602-s001.zip › materials-3504116-supplementary.pdf]

Supplementary Material

Changes in fluorescence of aquatic dissolved organic matter induced by plastic debris

Cristina L. Popa, Simona I. Dontu*, Dan Savastru and Elfrida M. Carstea*

National Institute of R&D for Optoelectronics, INOE2000, Atomistilor 409, Magurele, Ilfov, Romania Affiliation ; cristina.popa@inoe.ro, simona.dontu@inoe.ro, dsavas@inoe.ro, elfrida.carstea@inoe.ro
*Correspondence: elfrida.carstea@inoe.ro; simona.dontu@inoe.ro

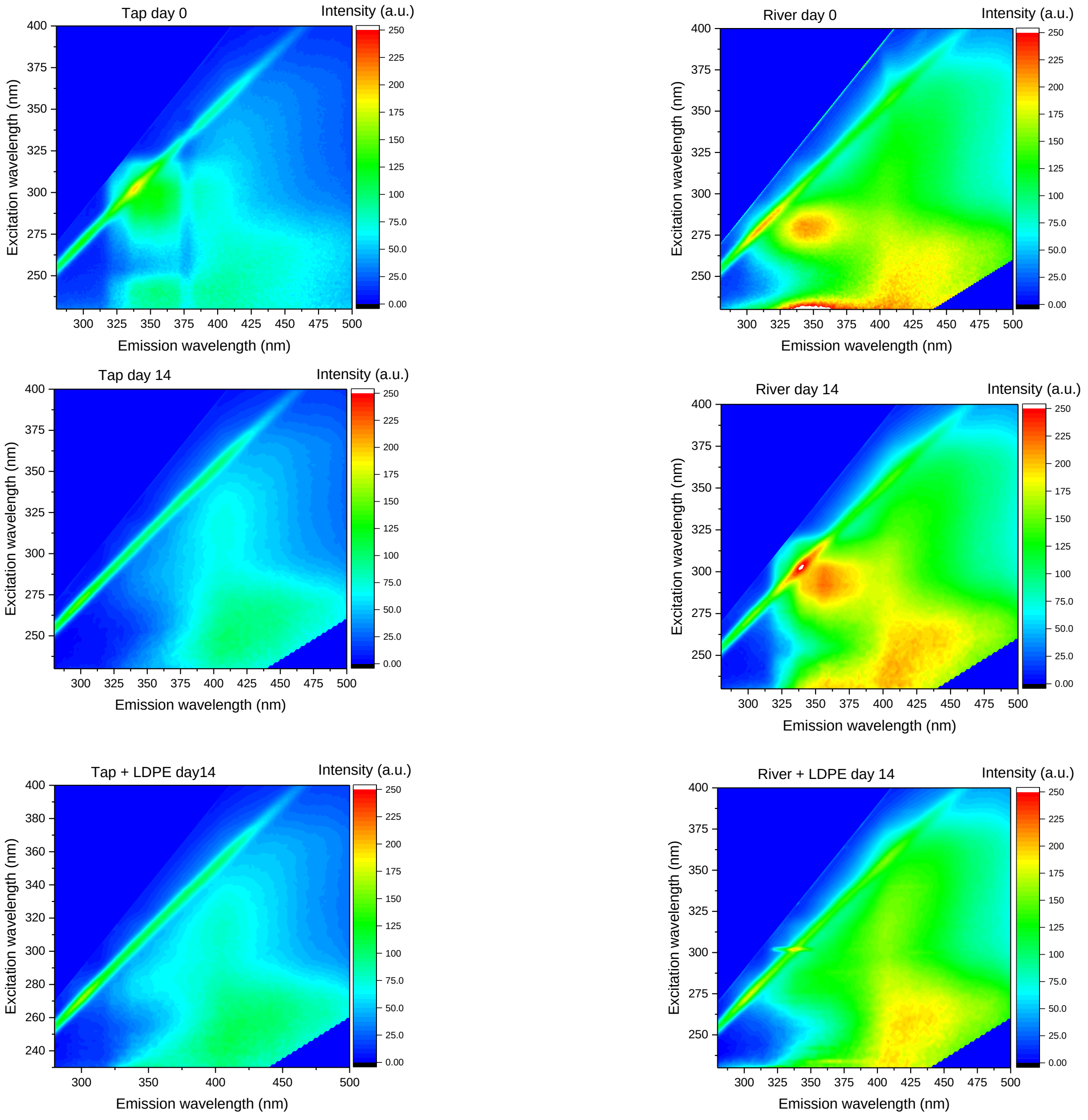

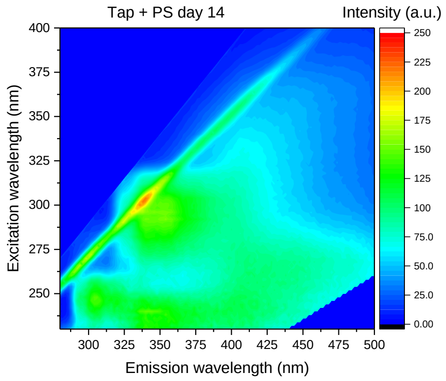

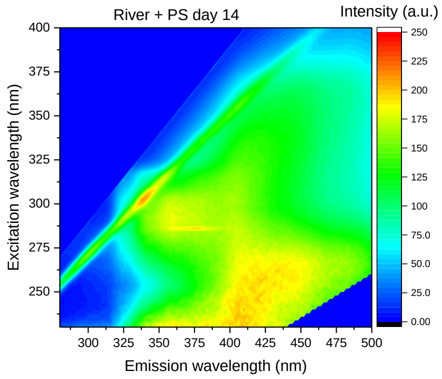

Figure S1. Fluorescence excitation-emission matrices for control day 0, control day 14, Tap+LDPE day 14, River+LDPE day 14, Tap +PS day 14, River+PS day 14.

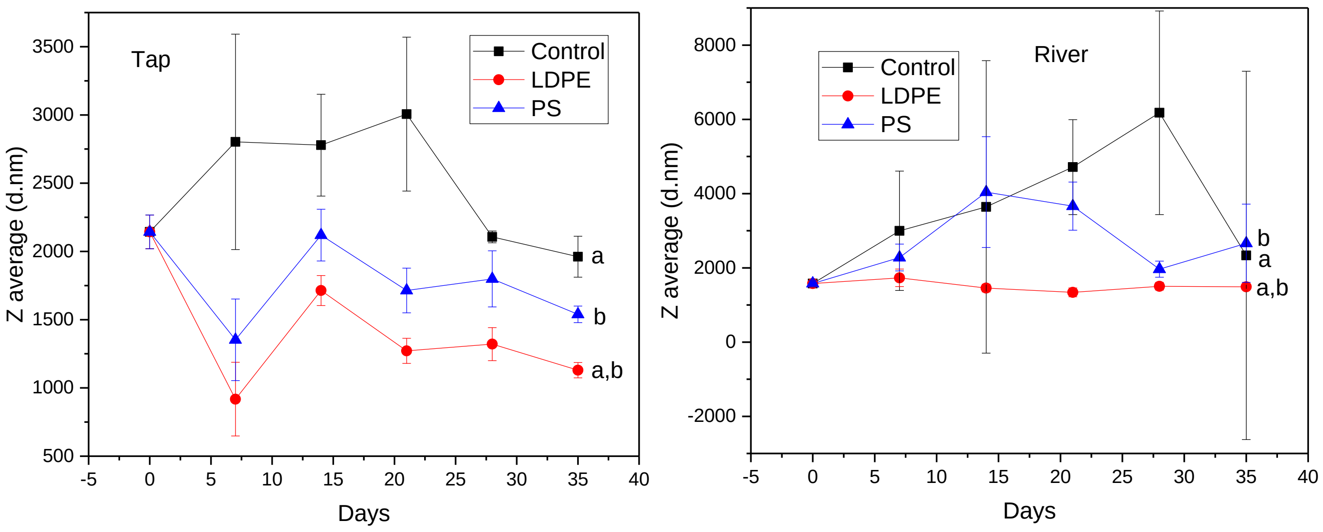

Figure S2. The average hydrodynamic size distribution for river and tap water samples. Parameters that present the same letter are not significantly different at $p < 0.01$.

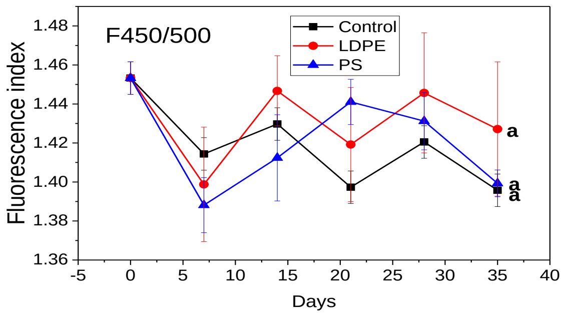

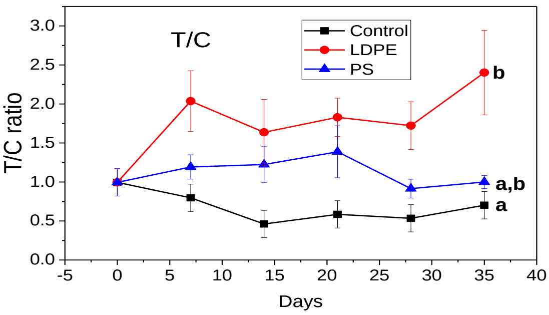

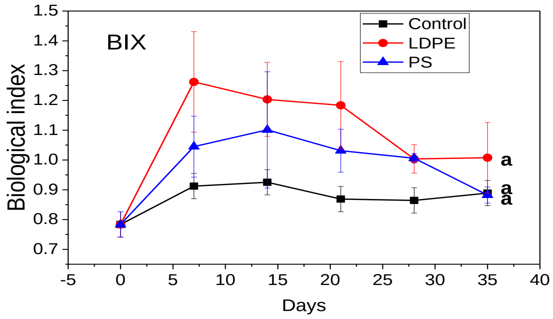

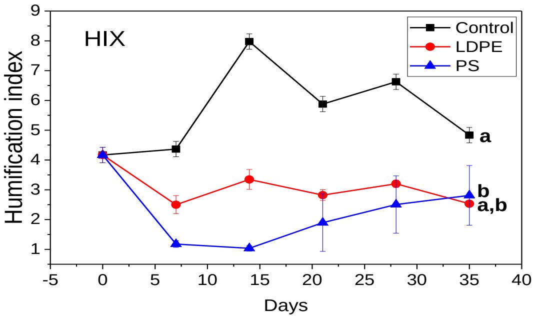

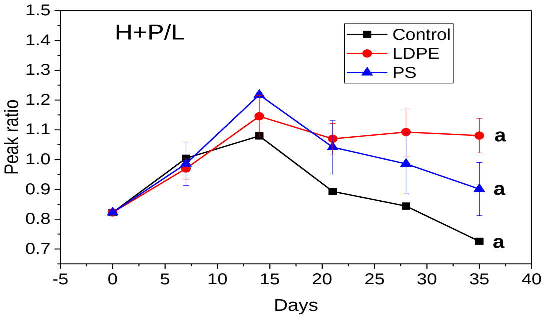

Tap water samples

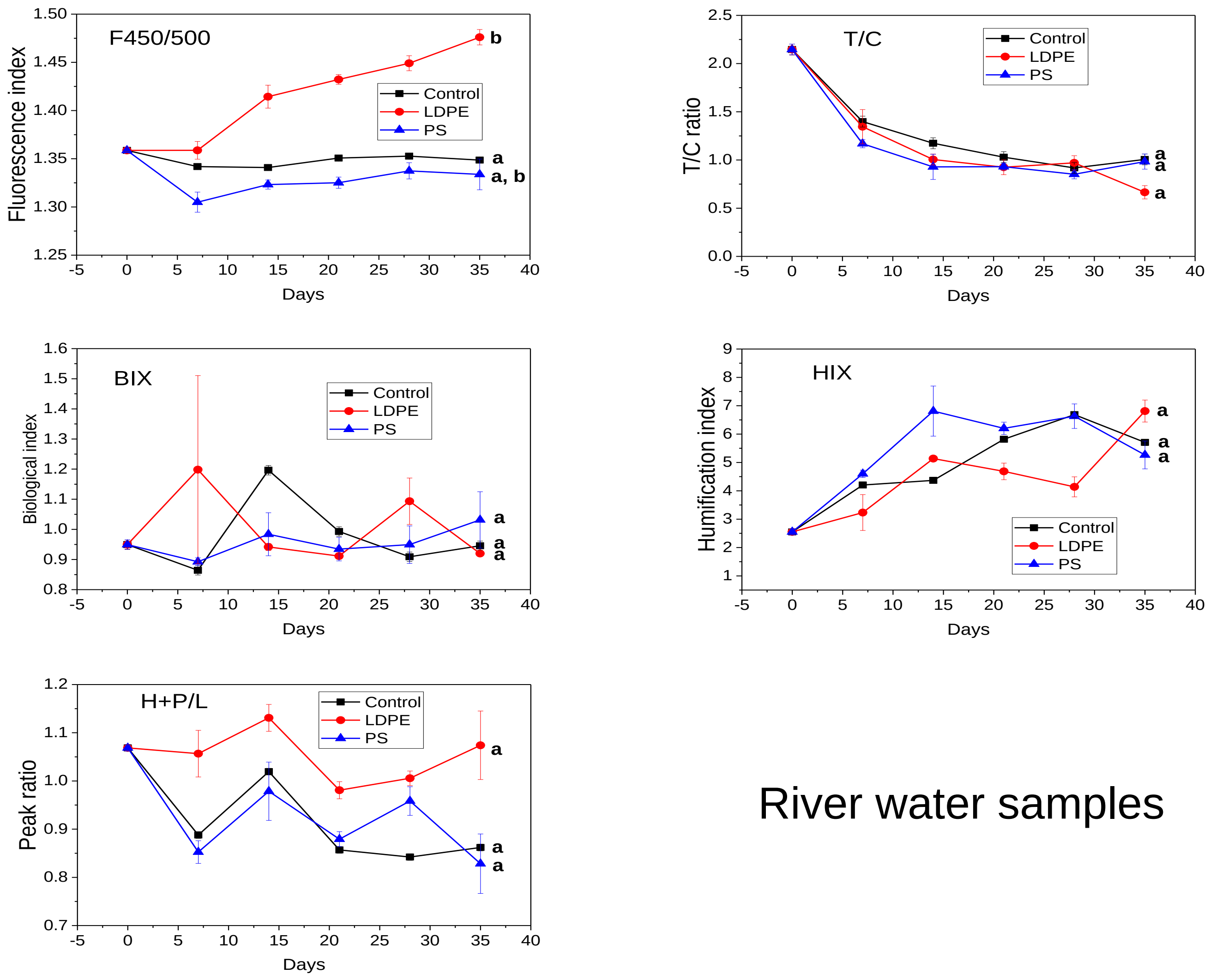

Figure S3. Changes in fluorescence indices values at tap and river samples exposed to PS and LDPE. The groups indicated with the same letter indicate that they are not significantly different ($p < 0.01$).

	Days		Peak B (R.U.)	Peak T230 (R.U.)	Peak T280 (R.U.)	Peak C (R.U.)	Peak A (R.U.)	Peak M (R.U.)	Peak DPBD (R.U.)	Peak UVX (R.U.)	Peak BPN (R.U.)	Peak H (R.U.)	Peak L (R.U.)	Peak P (R.U.)	(H + P)/L	HIX	BIX	F450/500	T/C	Z avg. (nm)	Cell abundance (cells/mL)	LNA/HNA
Tap	0	Control	-0.0209	0.0017	0.0098	0.0764	0.1825	0.0879	0.0347	0.0368	0.1164	0.0910	0.0914	-0.0158	0.8226	4.1673	0.7836	1.4533	0.9948	2143	5340	28.5909
	7	Control	-0.0276	-0.0027	0.0156	0.0600	0.0827	0.0756	0.0450	0.0379	0.1193	0.0798	0.0817	0.0022	1.0044	4.3661	0.9123	1.4144	0.7971	2803	8960	13.7907
		LDPE	0.0339	0.2551	0.1736	0.1165	0.2211	0.1827	0.0741	0.0519	0.2288	0.1576	0.2010	0.0375	0.9700	2.4982	1.2621	1.3987	2.0363	918	44960	2.3623
		PS	0.1044	0.0889	0.0723	0.0994	0.1284	0.1079	0.0489	0.0416	0.1525	0.1055	0.1241	0.0169	0.9862	1.1783	1.0449	1.3881	1.1925	1353	1740	60.7857
	14	Control	0.0126	0.0374	0.0434	0.0843	0.1709	0.1113	0.0731	0.0528	0.1816	0.1307	0.1376	0.0178	1.0795	7.9753	0.9253	1.4297	0.4612	2779	110980	0.6724
		LDPE	0.0620	0.2961	0.1816	0.1030	0.2628	0.1927	0.1006	0.0647	0.2630	0.1833	0.2170	0.0652	1.1454	3.3465	1.2033	1.4467	1.6363	1714	18460	15.2759
		PS	0.2201	0.1763	0.0994	0.0834	0.1772	0.1392	0.0737	0.0536	0.1844	0.1331	0.1463	0.0451	1.2176	1.0346	1.1011	1.4124	1.2237	2119	39300	48.9000
	21	Control	0.0357	0.0842	0.0577	0.0815	0.1745	0.1199	0.0695	0.0520	0.1821	0.1228	0.1540	0.0148	0.8932	5.8778	0.8687	1.3973	0.5853	3006	672360	5.9592
		LDPE	0.0997	0.3643	0.2291	0.1093	0.2891	0.2261	0.1065	0.0697	0.2776	0.2053	0.2642	0.0774	1.0699	2.8215	1.1837	1.4192	1.8293	1272	33400	22.6053
		PS	0.1896	0.2267	0.0944	0.0817	0.2795	0.1289	0.0698	0.0512	0.1845	0.1385	0.1729	0.0417	1.0417	1.8959	1.0313	1.4410	1.3872	1714	256660	4.9118
	28	Control	0.0252	0.0394	0.0223	0.0821	0.1685	0.1015	0.0659	0.0524	0.1693	0.1160	0.1520	0.0123	0.8441	6.6251	0.8642	1.4205	0.5347	2107	561700	9.4516
		LDPE	0.0942	0.3088	0.1636	0.1130	0.2808	0.1929	0.1125	0.0758	0.2794	0.1993	0.2550	0.0794	1.0925	3.2011	1.0037	1.4457	1.7225	1321	111220	19.0000
		PS	0.1454	0.1070	0.0654	0.0815	0.1788	0.1100	0.0699	0.0530	0.1830	0.1275	0.1667	0.0368	0.9856	2.5043	1.0059	1.4312	0.9150	1799	449840	4.4064
	35	Control	-0.0080	-0.0698	-0.1057	0.0774	0.1241	-0.0315	0.0622	0.0508	0.1590	0.0837	0.1262	0.0078	0.7256	4.8344	0.8888	1.3958	0.7022	1961	590120	4.2970
		LDPE	0.1777	0.3728	0.1052	0.1360	0.2966	0.1015	0.1162	0.0759	0.2668	0.1855	0.2528	0.0877	1.0806	2.5305	1.0076	1.4271	2.4020	1130	750340	64.9286
		PS	0.0993	-0.0174	-0.0864	0.0789	0.1312	-0.0247	0.0664	0.0530	0.1626	0.0870	0.1278	0.0282	0.9012	2.8114	0.8824	1.3993	0.9994	1539	574400	2.0061
River	0	Control	0.1183	0.3275	0.2077	0.1529	0.3114	0.1979	0.1264	0.1098	0.2902	2.5513	0.9495	0.2430	0.3130	0.0914	1.0686	1.3587	2.1464	1576	425120	14.0820
	7	Control	0.0407	0.2468	0.1532	0.1908	0.3143	0.2392	0.1101	0.1029	0.3100	4.2049	0.8642	0.2445	0.3094	0.0302	0.8878	1.3419	1.3984	2998	55320	20.7632
		LDPE	0.0690	0.2860	0.3056	0.2217	0.3298	0.3562	0.1100	0.1027	0.3047	3.2340	1.1984	0.2673	0.3142	0.0647	1.0566	1.3587	1.3454	1731	149180	33.6000
		PS	0.0304	0.1877	0.1307	0.1874	0.3140	0.2307	0.1083	0.1000	0.2968	4.6002	0.8926	0.2346	0.2978	0.0193	0.8525	1.3050	1.1690	2278	67560	21.3171
	14	Control	0.0554	0.3180	0.3181	0.2120	0.3860	0.3601	0.1384	0.1244	0.3794	4.3671	1.1964	0.3245	0.3597	0.0422	1.0194	1.3409	1.1736	7492	78420	19.0938
		LDPE	0.1078	0.2535	0.2090	0.1999	0.3685	0.2890	0.1471	0.1299	0.3547	5.1379	0.9417	0.3035	0.3423	0.0836	1.1309	1.4144	1.0048	1455	48320	15.1951
		PS	0.0458	0.2226	0.1872	0.1892	0.3573	0.2805	0.1436	0.1191	0.3482	6.8116	0.9838	0.2918	0.3361	0.0371	0.9786	1.3232	0.9289	6029	45800	17.3438
	21	Control	0.0671	0.2678	0.1941	0.1837	0.3806	0.2800	0.1285	0.1158	0.3562	5.8195	0.9930	0.2888	0.3773	0.0346	0.8569	1.3508	1.0293	4714	62440	31.3158
		LDPE	0.0946	0.2199	0.1805	0.1933	0.3699	0.2873	0.1421	0.1225	0.3319	4.6850	0.9113	0.2846	0.3540	0.0626	0.9808	1.4322	0.9250	1341	46040	21.0345
		PS	0.0692	0.2361	0.1662	0.1788	0.3680	0.2675	0.1342	0.1154	0.3424	6.2030	0.9345	0.2808	0.3576	0.0336	0.8792	1.3251	0.9309	3661	46040	72.4286
	28	Control	0.0539	0.1927	0.1226	0.1748	0.3684	0.2609	0.1239	0.1168	0.3462	6.6856	0.9090	0.2779	0.3613	0.0264	0.8422	1.3527	0.9180	6178	68800	28.8571
		LDPE	0.0760	0.2178	0.2315	0.2029	0.3615	0.2824	0.1422	0.1241	0.3285	4.1400	1.0934	0.2933	0.3431	0.0517	1.0056	1.4491	0.9706	1503	29720	13.3488
		PS	0.0490	0.1708	0.1294	0.1786	0.3360	0.2485	0.1327	0.0262	0.3304	6.6315	0.9494	0.2777	0.3197	0.0287	0.9584	1.3374	0.8519	1964	42540	55.7778
	35	Control	0.0286	0.0802	0.0003	0.1750	0.3155	0.1266	0.1215	0.1167	0.3253	5.7076	0.9456	0.2375	0.2968	0.0183	0.8620	1.3486	1.0062	7286	104560	70.5556
		LDPE	0.0057	0.0278	-0.0019	0.2341	0.3212	0.1776	0.1671	0.1468	0.3515	6.8129	0.9201	0.2906	0.3022	0.0339	1.0740	1.4761	0.6651	1490	51980	24.7600
		PS	0.0277	0.0799	0.0333	0.1790	0.3110	0.1405	0.1292	0.1115	0.3230	5.2645	1.0316	0.2374	0.3087	0.0183	0.8283	1.3338	0.9833	2661	45740	55.0833
